# Three-Dimensional (3D) Printing in Surgical Pathology: The Design of a Novel Grossing Tool to Aid Staple Removal in Pulmonary Pathology Excision Specimens

**DOI:** 10.7759/cureus.77714

**Published:** 2025-01-20

**Authors:** Panagiotis Kousidis, Nikolaos Barbetakis, Demetrios Paliouras, Despoina Masmanidou, Anastasia Nikolaidou

**Affiliations:** 1 Pathology, Theageneio Anticancer Hospital of Thessaloniki, Thessaloniki, GRC; 2 Thoracic Surgery, Theageneio Anticancer Hospital of Thessaloniki, Thessaloniki, GRC

**Keywords:** 3d, 3d printing, grossing, macroscopic examination, margin, nsclc, pulmonary pathology, segmentectomy, stapler, wedge resection

## Abstract

Pathologists or laboratory technicians who perform a gross examination of lung specimens may, under the pressure of performing a frozen section or in a less urgent setting when selecting permanent sections, encounter a specimen with a lesion close to a stapled surgical margin. Performing a perpendicular section from the lesion to the closest surgical margin is often necessary, and the technique of shaving the staple line from the specimen before sectioning may yield suboptimal results in margin assessment. It is widely accepted that staple removal is a tedious task, both time-consuming and, more importantly, with a high risk of damaging the stapled tissue and causing difficulty in its precise microscopic evaluation. With regard to three-dimensional (3D) printing, the emergence of software for 3D design, with easy-to-learn interfaces, available at no monetary cost, as well as the ability to have one's designs produced and delivered swiftly and at low cost through regional stores, has bridged the gap between conception and implementation of novel ideas in several sectors of medicine. A grossing tool was designed and 3D printed, with the aim of facilitating the procedure of staple removal by reducing the time required and the tissue damage caused. The tool was tested on test material created from synthetic sponges, with the goal of simulating the physical properties of lung parenchyma. The variables measured were time for removal per staple and estimated test material damage after removal of each staple. Two techniques for staple removal were used: the "pinch-and-pull" technique of vertically pulling the staple and the "push-through" technique of carefully lifting the staple by pushing the forceps perpendicularly underneath the staple. The results showed that the use of the tool is superior, with improvement for both variables in both techniques. Our report aims to showcase this novel grossing tool, present the approach to its creation, analyze relevant medical literature, and also highlight the ease of implementation and future prospects of 3D printed designs in medical education and practice.

## Introduction

Grossing of lung excision specimens, either in the setting of evaluation for frozen sections or post-fixation selection of permanent sections, is common for pathologists and grossing personnel practicing in hospitals in which thoracic surgery operations are performed. Especially regarding small peripheral tumors, the choice of sublobar resection versus lobectomy is a critical contemporary issue in the management of resectable non-small cell lung cancer (NSCLC) [1]. In excision specimens, the decision of the need for further excision or potential lobectomy after an initial sublobar resection can be intraoperatively assessed via a frozen section, in which a very high level of precision is required [2]. Especially in this setting, accurate gross and microscopic examination is crucial, as misinterpretation of small differences in margins can have great prognostic implications [1]. Major grossing manuals often refer to the consideration of staple removal in tumors that are positioned close to the surgical margin, highlighting the risks of causing damage to stapled tissue [3] and the extensive time required [4]. Optimizing this procedure can aid in performing rapid and accurate frozen section examination, but also in the precision of permanent sections, allowing for a more effective appreciation of surgical margins.

With this goal, a three-dimensional (3D) model of a new grossing tool was designed. We also constructed test material from the synthetic sponge, upon which the tool was tested. Measurements were performed using two different techniques of staple removal, with and without concurrent use of the tool, for two variables related to its use. We analyze the final results and discuss the ease of implementation and the future potential for 3D printing in medical education and practice.

## Technical report

**Figure 1:**
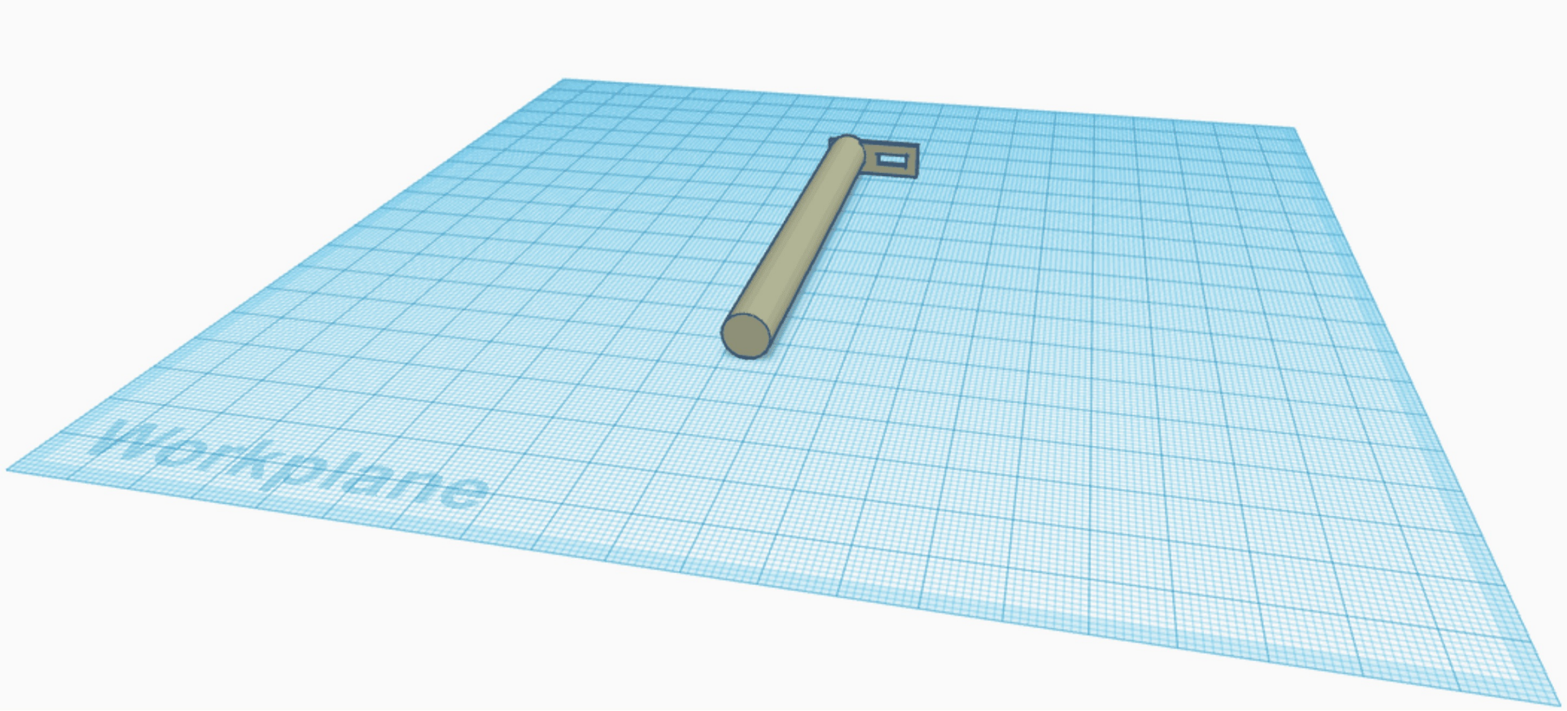
Picture of a three-dimensional (3D) model tool design. The image was created using the software application media interface (Autodesk TinkerCAD) [5]. The design of the grossing tool was finalized after having multiple previous prototypes three-dimensional (3D) printed and tested. The ease of design and low monetary cost for production facilitate experimentation with different prototypes. The body of the tool consists of an elongated, solid cylindrical handle. At its distal end, a thin rectangular structure protrudes radially, on which a smaller rectangular opening exists, through which the staple is manipulated. The straightforward and simple design, requiring no post-processing and being ready to use upon delivery, is essential, as the probability of error in its 3D printing is lower, the design is more easily reproducible, and the production cost is smaller. The use of plastic material allows for the flexibility of the protruding rectangle, which makes the use of the instrument easier and minimizes damage to the staple line upon staple removal.

A test material model was created, with the aim of simulating the mechanical properties of stapled lung tissue (mainly elasticity, compressibility, cohesive and tensile strength), while also maintaining the low cost of the study. Selected commercially available synthetic sponge consistency materials were used, in which metallic staples were applied, in a pattern and at a scale consistent with the encountered excised specimens of our institution, in which staplers with three parallel rows of staples were utilized. A 3D model of a new tool was designed (Figure 1), using 3D-design software available online at no cost (Autodesk TinkerCAD) [5].

**Figure 2:**
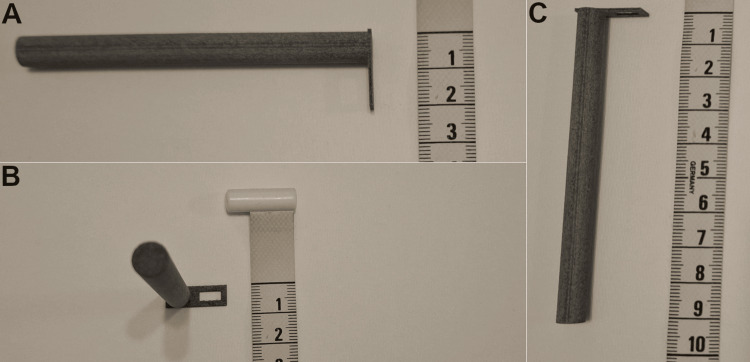
Final produced model of the grossing tool: main model. (A) Top view, showing the measurements of the diameter of the handle and the attached rectangle length; (B) Left view, showing the attached rectangle and the rectangle opening width; (C) Top view, rotated 90 degrees counterclockwise, showing the handle length and the attached rectangle thickness. Three-dimensional (3D) printing has become an affordable and rapid way to create working prototypes of tools and equipment in many sectors, including medicine. In the regional store we selected, the cost for production of a single prototype tool was equivalent to 16.27 USD, including value-added tax (24%). The prototypes were 3D printed with the technique of selective laser sintering (SLS), using nylon powder as material. The total time for printing and delivery was five working days.

**Figure 3:**
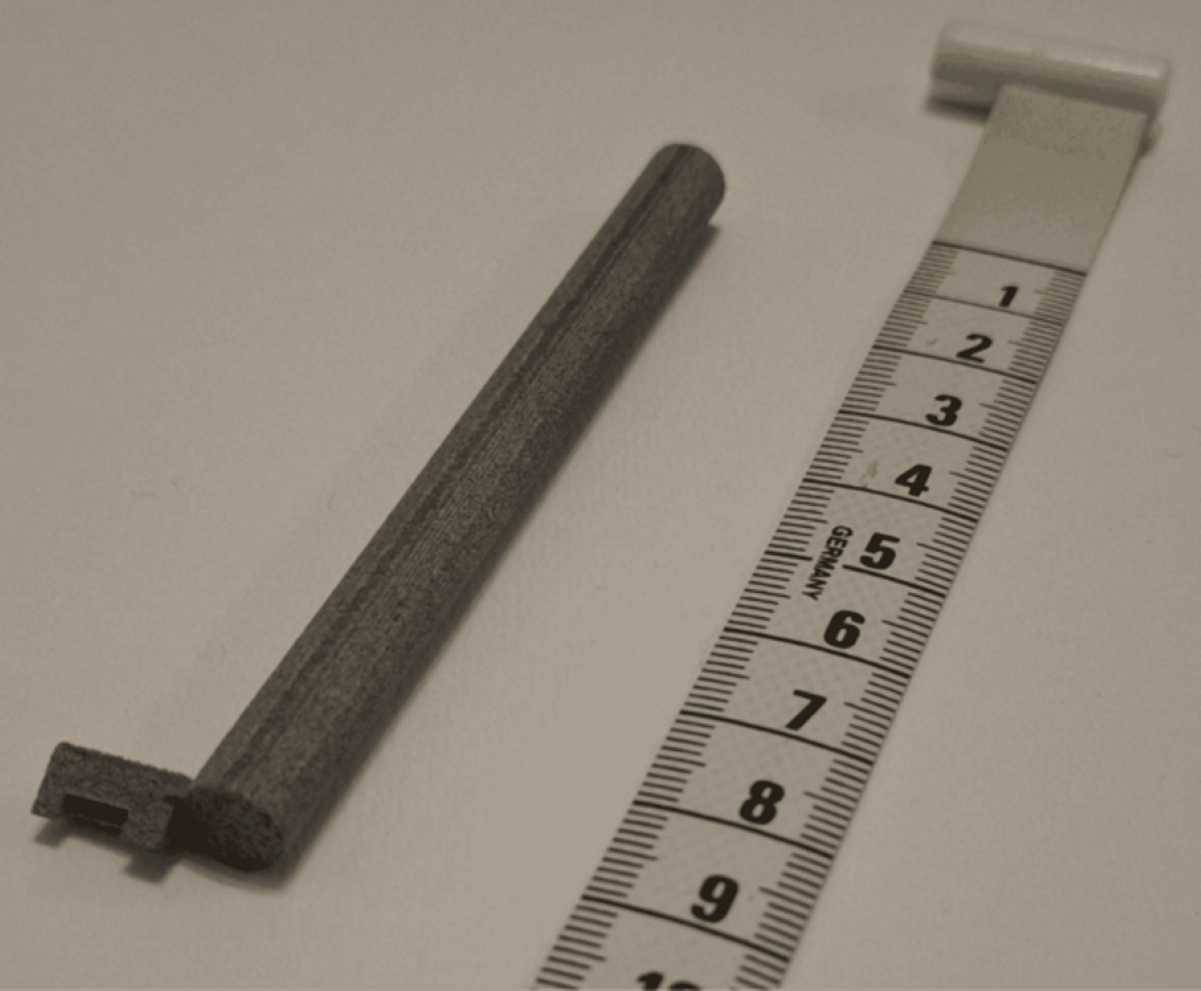
Prototype of a second model of the grossing tool: variant model. A variant model was created after the main model, which aids in removing staples in special situations where the relative anatomy of the staple line to the adjacent specimen has been altered, especially post-fixation. This variant model has a thicker, sturdier rectangular end, sharper edges protruding downwards, and a slightly smaller opening for accessing each staple.

The model was printed at a regional 3D printing laboratory. The main tool model (Figure 2), as well as a variant model (Figure 3), were produced. The variant model is useful for accessing deeply placed staples if a groove is formed between the proximal staple line and adjacent lung parenchyma due to tissue dynamics, occurring before or, more commonly, after fixation.

**Figure 4:**
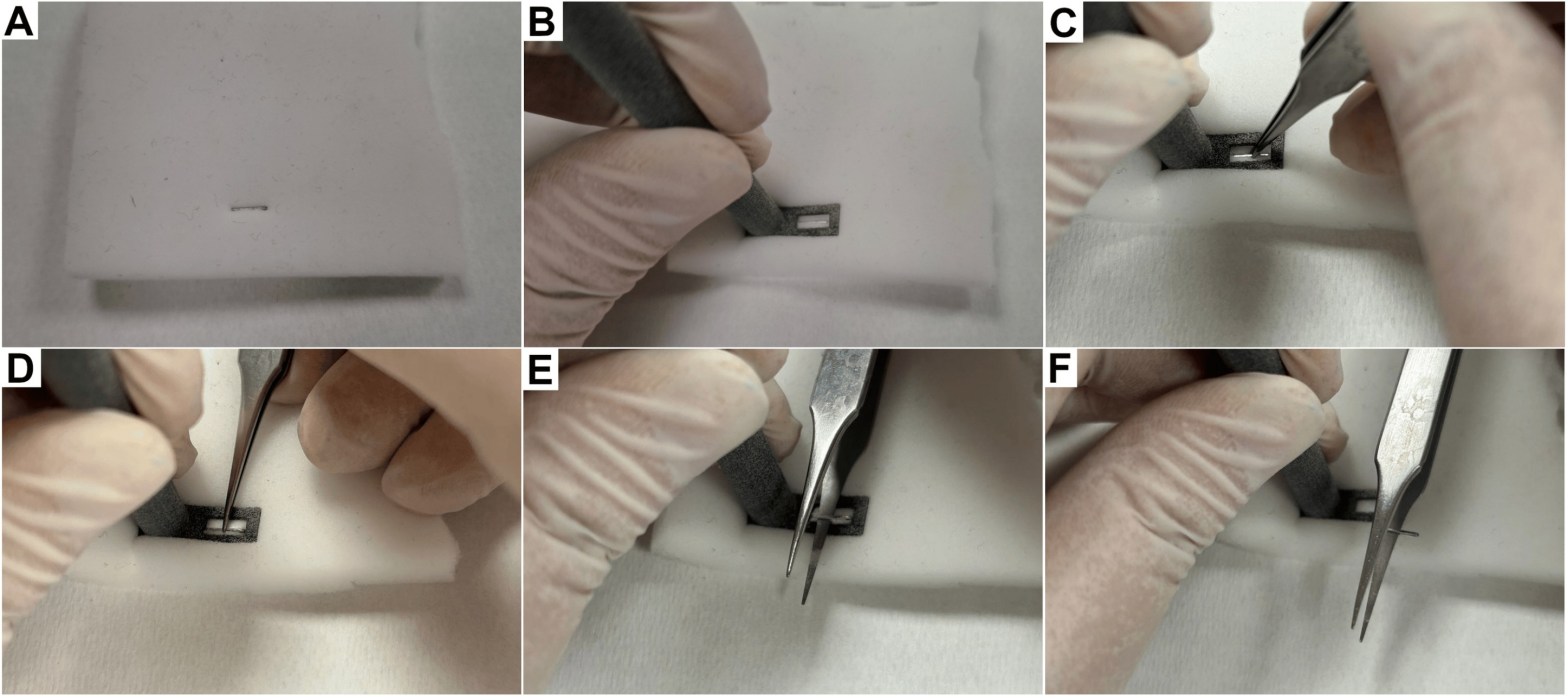
Steps for staple removal by using the main model of the designed grossing tool: the "push-through" technique. The staple (A) is held in place by applying downward pressure with the tool, whilst keeping the staple centered through the rectangular opening (B). The staple is caught with the very fine end forceps (C), which are then rotated in order to have one end pass underneath the staple (D). The forceps are then pushed toward the user instead of pulling immediately upwards (E). By using this technique, most staples will detach from the model without compromising model integrity (F).

The grossing tool aims to hold the lung tissue adjacent to the staple in place, maintaining integrity and preventing tears in the stapled portion of the specimen during staple removal by transmitting downward pressure and stabilizing the surrounding tissue, while the embedded staple is pulled through the opening with a set of very fine end forceps. The main model of the grossing tool was tested by the lead and senior author (P.K. and A.N., respectively) with the given goal of swift removal of staples with minimal damage to the test material. We performed separate measurements using two different techniques: the "pinch-and-pull" technique, in which the staples are grasped with the very fine end forceps and pulled vertically off the model, and the "push-through" technique, in which the very fine end forceps is used in a pinching and then rotating and pushing motion to remove the staple (Figure 4). These steps are continued until all of the desired staples are removed.

**Figure 5:**
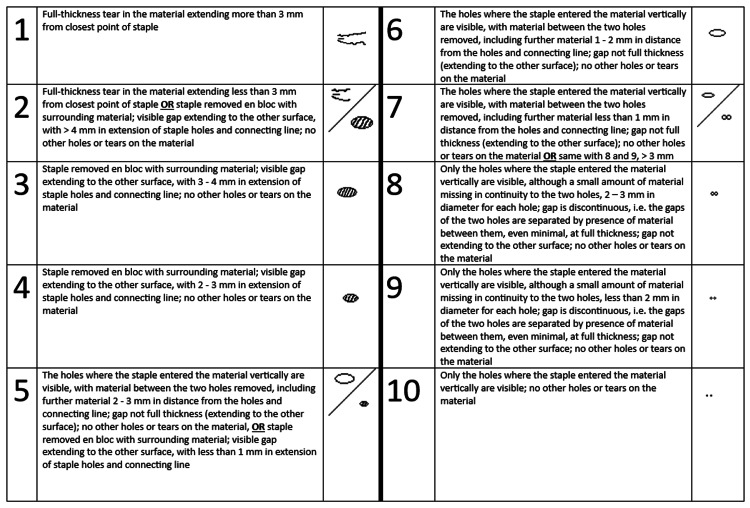
Scale for evaluation of test material damage after removal of each staple. A scale was improvised in order to accurately categorize measurements made for the damage caused during the removal of each staple. A small image accompanies the definition of each rank, in which shapes containing stripes indicate full-thickness loss of material, and shapes with solid white content indicate a non-full-thickness loss.

**Figure 6:**
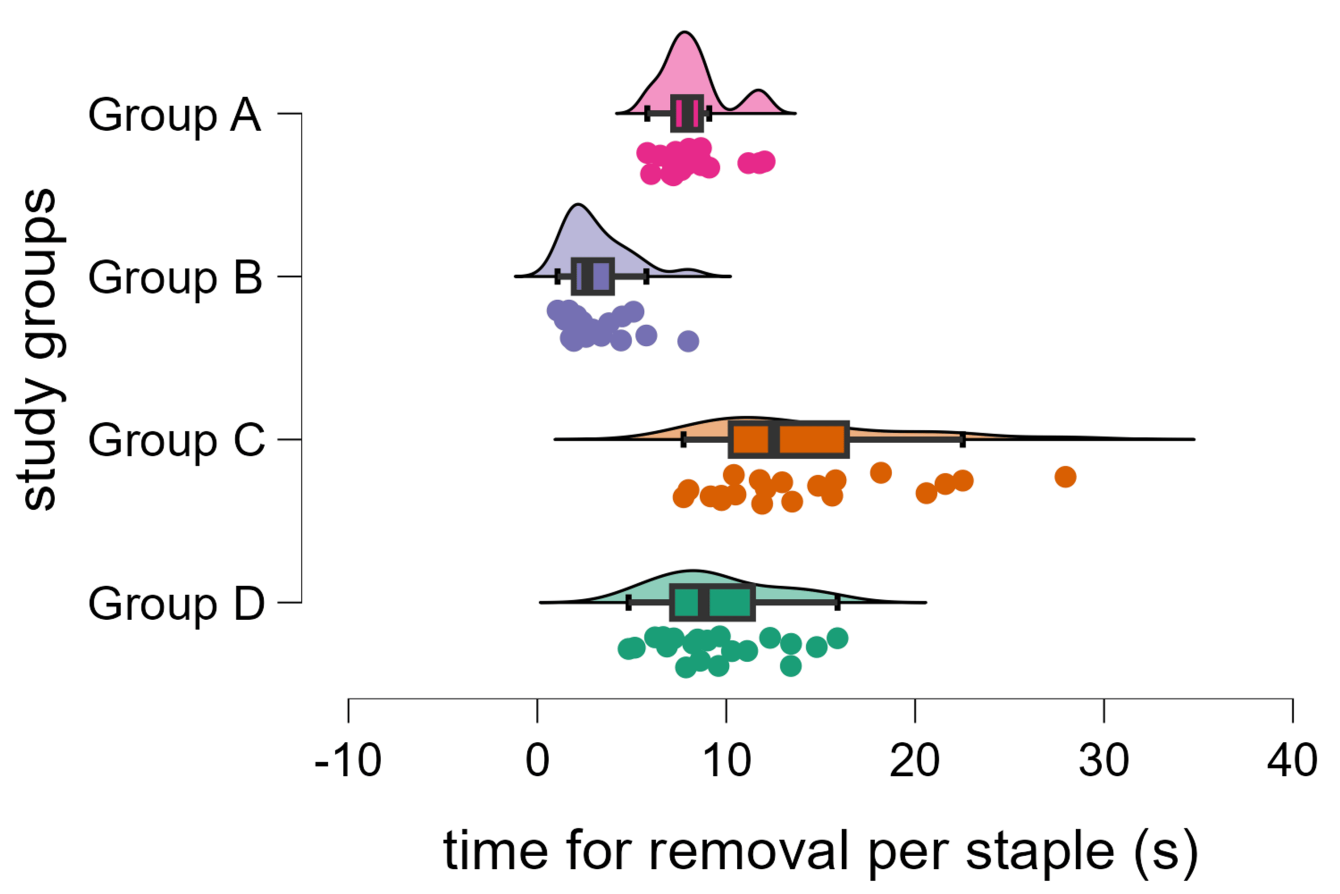
Plot of time for removal per staple, along the four examined groups. Raincloud plot of the time for staple removal (in seconds) for each of the four study groups. Group A: "pinch-and-pull" technique without use of the novel grossing tool, using a pair of 14-cm anatomic forceps and a pair of very fine end forceps; Group B: "pinch-and-pull" technique with use of the novel grossing tool and a pair of very fine end forceps; Group C: "push-through" technique without use of the novel grossing tool, using a pair of 14-cm anatomic forceps and a pair of very fine end forceps; Group D: "push-through" technique with use of the novel grossing tool and a pair of very fine end forceps.

**Figure 7:**
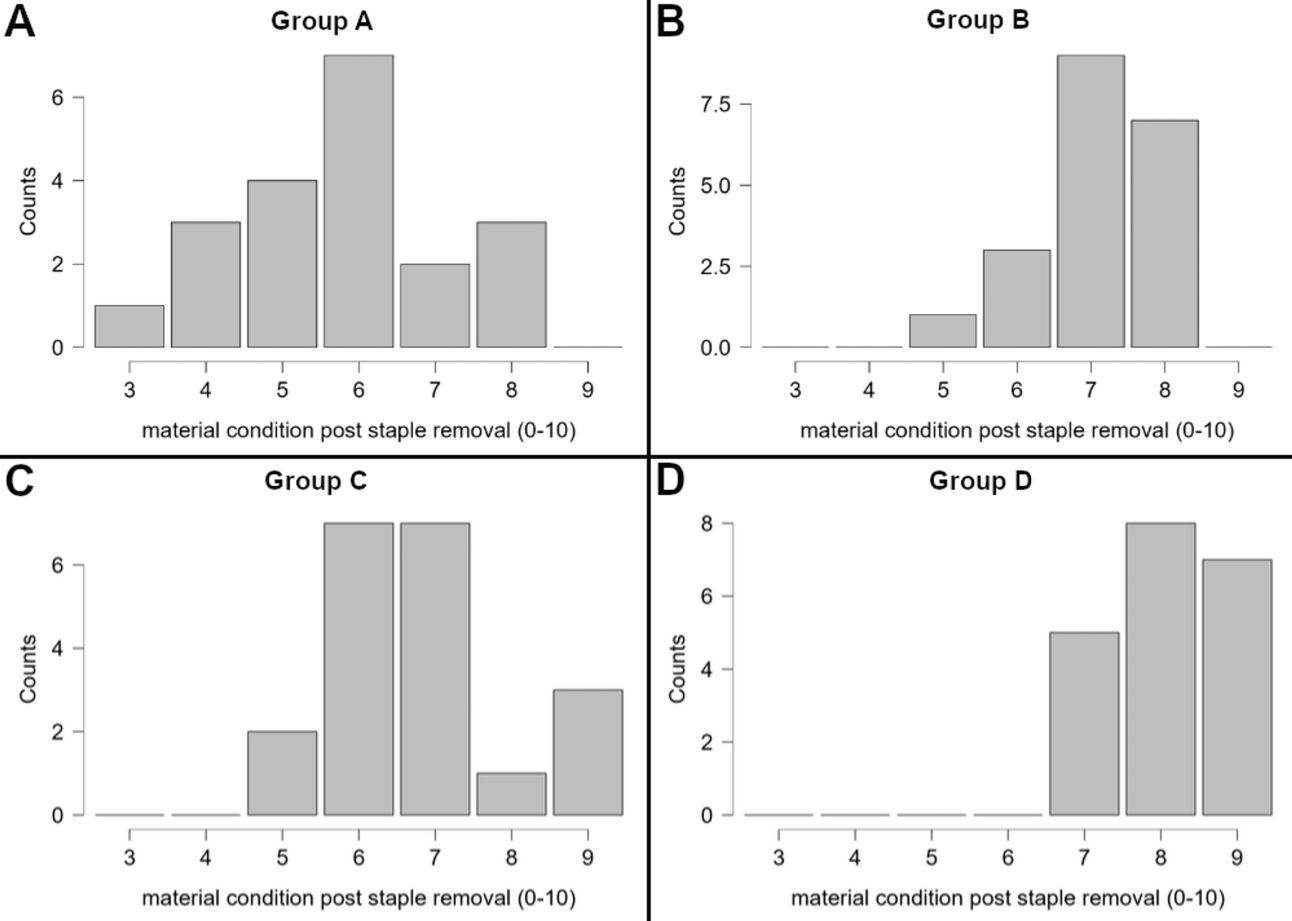
Plots of test material condition after staple removal, along the four examined groups. Bar plots (A-D), describing the value assigned from the scale (1-10) regarding the test material condition after staple removal for each of the four study groups. (A) Group A: "pinch-and-pull" technique without use of the novel grossing tool, using a pair of 14-cm anatomic forceps and a pair of very fine end forceps; (B) Group B: "pinch-and-pull" technique with use of the novel grossing tool and a pair of very fine end forceps; (C) Group C: "push-through" technique without use of the novel grossing tool, using a pair of 14-cm anatomic forceps and a pair of very fine end forceps; (D) Group D: "push-through" technique with use of the novel grossing tool and a pair of very fine end forceps.

In order to evaluate whether the novel grossing tool is superior to the removal of staples by the usual technique, we created four groups for statistical comparison. In the first group (Group A), staple removal was performed by using the "pinch-and-pull" technique, without the use of the novel grossing tool, using a pair of 14-cm anatomic forceps and a pair of very fine end forceps. In the second group (Group B), staple removal was performed with the same technique of "pinch-and-pull," but with the use of the novel grossing tool and a pair of very fine end forceps. In the third group (Group C), the "push-through" technique was used, with the use of a pair of 14-cm anatomic forceps and a pair of very fine end forceps, and in the fourth group (Group D), the "push-through" technique was used, with the use of the novel grossing tool and a pair of very fine end forceps. Measurements were made regarding time for removal per staple (in seconds, continuous variable) and estimated test material condition after removal of each staple (1-10, ordinal variable; Figure 5), with 20 measurements of each variable made for each of the four groups (Figures 6-7). The comparison of Group A versus Group B and Group C versus Group D was the main analysis, aiming to examine the potential superiority of using the grossing tool versus the usual tools for staple removal using a given removal technique. Evaluation of normality with the Shapiro-Wilk test showed that for the continuous variable of time for removal per staple, the null hypothesis was rejected for Group A and Group B (p < 0.05 for both groups), with a trend towards significance for Group C (p = 0.053), yet no rejection of the null hypothesis for Group D (p = 0.445). The Kruskal-Wallis test was used, and Dunn's pairwise multiple comparisons were applied with Holm's correction of p-value. Statistical significance was set at p < 0.05. Data analysis was performed by JASP, version 0.19.2.0 (Jeffreys's Amazing Statistics Program, Amsterdam, the Netherlands) [6].

Analysis of the data indicated that, for both techniques, using the novel tool resulted in a shorter time for removal per staple (Table 1) and higher test material condition after removal of each staple (Table 2). Some further observations were also made. Expectedly, the "pinch-and-pull" technique results in faster staple removal versus the "push-through" technique with the same tools used (Group A - Group C and Group B - Group D; Table 1). Importantly, the "push-through" technique with use of the novel tool was the superior group in higher test material condition post-staple removal against the other three groups, while the "pinch-and-pull" technique with use of the novel tool was the superior group against the others in shorter time for removal per staple.

**Table 1:** Results of the Kruskal-Wallis test on time for removal per staple, for each technique and tool group: Dunn's pairwise multiple comparisons. The two comparisons relative to the analysis of the potential superiority of use of the novel grossing tool are written in italics. The statistically significant results after the Holm correction (defined at p < 0.05) are written in bold. It must be noted that, in the interpretation of results, a positive z-value with a p-value <0.05 indicates a statistically significant longer time for staple removal in the first reported group against the second, with a negative z-value indicating a shorter time. Group A: "pinch-and-pull" technique without use of the novel grossing tool, using a pair of 14-cm anatomic forceps and a pair of very fine end forceps; Group B: "pinch-and-pull" technique with use of the novel grossing tool and a pair of very fine end forceps; Group C: "push-through" technique without use of the novel grossing tool, using a pair of 14-cm anatomic forceps and a pair of very fine end forceps; Group D: "push-through" technique with use of the novel grossing tool and a pair of very fine end forceps. df: degrees of freedom; CI: confidence interval; ε^2^: Kruskal-Wallis epsilon squared; z: z-statistic value of specific group comparison; W_i_: mean rank of the first group of comparison; W_j_: mean rank of the second group of comparison; r_rb_: value of rank biserial correlation (based on individual Mann-Whitney tests); p_bonf_: p-value after Bonferroni correction for multiple comparisons; p_holm_: p-value after Holm correction for multiple comparisons.

Time for removal per staple															
Kruskal-Wallis Test															
Factor		Statistic		df		p		Rank ε²		95% CI for Rank ε²: lower			95% CI for Rank ε²: upper		
Study groups		53.354		3		<0.001		0.675		0.599			0.779		
Dunn’s Post Hoc Comparisons - Study Groups															
Comparison		z		W_i_		W_j_		r_rb_		p		p_bonf_		p_holm_	
Group A - Group B		3.814		39.575		11.550		0.950		<0.001		<0.001		<0.001	
Group A - Group C		-3.358		39.575		64.250		0.810		<0.001		0.005		0.002	
Group A - Group D		-0.959		39.575		46.625		0.233		0.337		1.000		0.337	
Group B - Group C		-7.172		11.550		64.250		0.995		<0.001		<0.001		<0.001	
Group B - Group D		-4.773		11.550		46.625		0.950		<0.001		<0.001		<0.001	
Group C - Group D		2.398		64.250		46.625		0.570		0.016		0.099		0.033	

**Table 2:** Results of the Kruskal-Wallis test on estimated test material condition after staple removal, for each technique and tool group: Dunn's pairwise multiple comparisons. The two comparisons relative to the analysis of the potential superiority of use of the novel grossing tool are written in italics. The statistically significant results after the Holm correction (defined at p < 0.05) are written in bold. It must be noted that, in the interpretation of results, a positive z-value with a p-value <0.05 indicates a statistically significant higher material condition after staple removal in the first reported group against the second, with a negative z-value indicating a lower condition on the applied scale. Group A: "pinch-and-pull" technique without use of the novel grossing tool, using a pair of 14-cm anatomic forceps and a pair of very fine end forceps; Group B: "pinch-and-pull" technique with use of the novel grossing tool and a pair of very fine end forceps; Group C: "push-through" technique without use of the novel grossing tool, using a pair of 14-cm anatomic forceps and a pair of very fine end forceps; Group D: "push-through" technique with use of the novel grossing tool and a pair of very fine end forceps. df: degrees of freedom; CI: confidence interval; ε^2^: Kruskal-Wallis epsilon squared; z: z-statistic value of specific group comparison; W_i_: mean rank of the first group of comparison; W_j_: mean rank of the second group of comparison; r_rb_: value of rank biserial correlation (based on individual Mann-Whitney tests); p_bonf_: p-value after Bonferroni correction for multiple comparisons; p_holm_: p-value after Holm correction for multiple comparisons.

Material condition post-staple removal															
Kruskal-Wallis Test															
Factor		Statistic		df		p		Rank ε²		95% CI for Rank ε²: lower			95% CI for Rank ε²: upper		
Study groups		29.994		3		<0.001		0.380		0.244			0.586		
Dunn's Post Hoc Comparisons - Study Groups															
Comparison		z		W_i_		W_j_		r_rb_		p		p_bonf_		p_holm_	
Group A - Group B		-2.861		22.250		42.750		0.570		0.004		0.025		0.017	
Group A - Group C		-1.943		22.250		36.175		0.415		0.052		0.312		0.104	
Group A - Group D		-5.384		22.250		60.825		0.840		<0.001		<0.001		<0.001	
Group B - Group C		0.918		42.750		36.175		0.228		0.359		1.000		0.359	
Group B - Group D		-2.523		42.750		60.825		0.573		0.012		0.070		0.035	
Group C - Group D		-3.440		36.175		60.825		0.620		<0.001		0.003		0.003	

## Discussion

In the management of resectable NSCLC, sublobar resections are preferred for lesions equivocal for malignancy, for lesions in patients with limited physiological reserve, or for lesions with mainly ground glass opacity pattern [1], provided that there is no evidence of positive lymph nodes [7]. Multiple studies have compared outcomes in lesions ≤2 cm in diameter, peripheral NSCLC [1], with recent data showing, possibly, at least noninferiority versus lobectomy in selected cases [7,8]. Frozen section examination is often employed to confirm malignancy in equivocal lesions and to confirm clear surgical margins or indicate the need for further synchronous excision or metachronous adjuvant treatment. Patient prognosis is highly dependent on precise frozen section analysis [2]. Optimally, achieving at least a 2 cm or equal-to-tumor-diameter parenchymal margin is generally the goal in sublobar resections [8], as studies have shown that margin positivity is inversely related to tumor-surgical margin distance, with a 2 cm distance [1] or equal-to-tumor-diameter [9] considered ultimately safe in the majority of cases. An interesting technique for cytological examination of the surgical margin has also been described [10].

Grossing of wedge resections is critical, as small differences in margins have great prognostic implications [1]. Margin assessment is complicated by the fact that there are different types of measurable resection margins in wedge resections, potentially causing confusion and requiring great care in measuring and reporting [11,12], while non-removal of staples can also alter tissue dynamics and result in potentially overestimating margin distance [12]. There is also a probability of the presence of non-continuous disease or atypical adenomatous hyperplasia (AAH), which can be missed if all the stapled margin tissue is trimmed [1].

**Figure 8:**
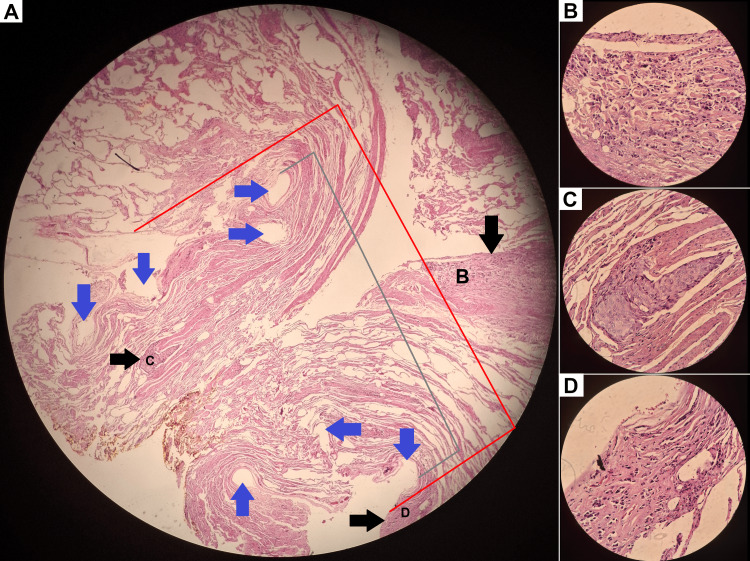
Illustrative case of handling of resection margins. A patient with a history of invasive breast carcinoma of no special type (IBC-NST), having previously undergone complete remission, presented with multiple pulmonary nodules. A wedge resection was performed. On gross examination of the excision specimen, the true margin (yellow ink) was preserved. The indentations of the surgical clips are visible (blue arrows), with three foci of neoplasia in the current section (black arrows). A stapler with three parallel staple lines was used; however, four indentations are visible on one side due to the tissue mechanics of the specific section. As the largest lesion (3 o'clock) (B) was larger than visible in the current field of view and was palpable and situated close to the true margin, we chose to preserve the staple line. Interestingly, two more foci were found on microscopic examination (5 o'clock and 8 o'clock) (C, D). An approach with trimming of the staple line would most likely result in missing the focus closest to the margin (C) altogether, with an indication of R0 excision (grey line), or potentially in reaching an erroneous final result of R1 excision (red line). The final pathologic diagnosis was metachronous pulmonary metastasis of IBC-NST. Another interesting observation is that the changes in anatomy caused by the staples remained, even though the staples were removed, presumably due to fixation. (A): H&E 4x; (B)-(D): H&E 40x. De-identified slide photographs, staples removed without use of novel tool pending authorization.

Major grossing manuals often refer to the consideration of staple removal in the gross examination of specimens with tumors that are positioned close to the surgical margin [3,4]. Different approaches have been described, including universal removal of the staple line from the specimen with the inking of the cut surfaces (and adding an estimated length of staple line) [3], as the stapled tissue is, in some manuals, deemed unsalvageable [13], and sectioning of the specimen with consideration of staple removal if the tumor is found in close proximity to the surgical margin. A method for staple removal on a section concurrently selected for frozen section examination has also been described. After placement of the section in embedding medium and freezing, prior to cryostat sectioning, attempts can be made to access and remove the staples, since the very low temperature of the section increases its integrity [3], a technique requiring high skill. The rare need to remove the staples from an already sectioned staple line is also highly technically challenging. It is thus evident that, in the setting of sublobar resections, especially wedge resections for early NSCLC, the thoracic surgeon's aim for preserving lung parenchyma results inevitably in specimens with a trend toward modest size, with a rising percentage of its volume being the potential neoplastic lesion. Staple presence can skew normal anatomy and measured diameter of the lesion and macroscopic lesion-to-margin distance, but also affect the accuracy of microscopic examination if the stapled tissue, and thus true margin, is damaged upon staple removal or not submitted, possibly leading to diagnostic error (Figure 8). These observations stress the importance of an effective adjunct tool in performing the staple removal procedure. This grossing tool has a low-cost, simple yet effective design and serves as a springboard for continuing our research on 3D printed tools in pathology.

Our novel grossing tool has led to statistically significant improvement in both time for staple removal and test material condition after removal of each staple, applicable to both the techniques of "push-through" and "pinch-and-pull." The low cost and ease of production suggest that it is a cost-effective and practical addition to the armamentarium of pathology grossing station equipment. An interesting future direction is its potential use in the grossing of stapled specimens from gynecological or general surgery. Modified, custom versions of the tool could also be created for use on small and delicate specimens in pediatric pathology.

Our report adds to the well-established potential of 3D printing in medicine, including the field of pathology [14-17]. In recent years, the body of literature has been continuously growing. More specifically, 3D printing techniques have been utilized for the creation of anatomic prototypes for the planning of patient-specific surgical operations, for the creation of patient-specific prostheses, and for medical teaching and education [18,19]. Regarding medical instruments, both the on-demand production of conventional surgical instruments in special environments, such as space missions [18], as well as the production of novel designs of non-conventional instruments, in a similar manner to ours, have been explored [20-22]. There is great potential for further integration of these techniques in medical practice in the future.

Testing on non-biological, synthetic sponge material, aiming to simulate the mechanics of lung tissue, with staples not identical to the ones used in our institution's excision specimens, runs the risk of the results being not completely applicable to stapled excision specimens. We hope to continue development and produce a tool from medical-grade acrylonitrile butadiene styrene (ABS) plastic, with a version possibly including a light-emitting diode (LED) for illumination of the working empty rectangular space of the tool, for more extensive testing in the setting of gross examination in pathology, after appropriate Institutional Review Board (IRB) approval.

## Conclusions

We believe that this new tool will be helpful to both pathologists and non-pathologist grossing personnel in order to optimize ultra-accurate diagnosis in specimens with tumors situated close to or on the staple line. Creating a new tool, however, for wide commercial circulation requires rigorous research and development from multidisciplinary scientist teams, as well as appropriate approval for extensive testing before results that can be generalized to everyday medical practice are reached. Our study aims to emphasize the closing of the gap between the conception of novel ideas and their initial application in medicine, in the field of medical equipment and tools, through the use of 3D printing techniques. Designing and 3D printing new instruments is no longer limited to specialists. The creation of this novel grossing tool underscores the potential for technology-driven innovation, including the field of 3D printing, to enhance diagnostic workflows, improve patient outcomes, and advance the field of pathology.
